# Research progress on and molecular mechanism of vacuum sealing drainage in the treatment of diabetic foot ulcers

**DOI:** 10.3389/fsurg.2024.1265360

**Published:** 2024-02-23

**Authors:** Yongpan Lu, Dejie Zhao, Guoqi Cao, Siyuan Yin, Chunyan Liu, Ru Song, Jiaxu Ma, Rui Sun, Zhenjie Wu, Jian Liu, Peng Wu, Yibing Wang

**Affiliations:** ^1^ First Clinical Medical College, Shandong University of Traditional Chinese Medicine, Jinan, China; ^2^ Jinan Clinical Research Center for Tissue Engineering Skin Regeneration and Wound Repair, The First Affiliated Hospital of Shandong First Medical University & Shandong Provincial Qianfoshan Hospital, Jinan, China; ^3^ Department of Vascular Surgery, Affiliated Hospital of Shandong University of Traditional Chinese Medicine, Jinan, China; ^4^ Department of Plastic Surgery, Shandong Provincial Qianfoshan Hospital, Shandong University, Jinan, China; ^5^ Department of Plastic Surgery, The First Affiliated Hospital of Shandong First Medical University & Shandong Provincial Qianfoshan Hospital, Jinan, China

**Keywords:** vacuum sealing drainage, trauma repair, diabetic foot ulcers, molecular mechanism, clinical treatment

## Abstract

Diabetic foot ulcers (DFUs) are common chronic wounds and a common complication of diabetes. The foot is the main site of diabetic ulcers, which involve small and medium-sized arteries, peripheral nerves, and microcirculation, among others. DFUs are prone to coinfections and affect many diabetic patients. In recent years, interdisciplinary research combining medicine and material science has been increasing and has achieved significant clinical therapeutic effects, and the application of vacuum sealing drainage (VSD) in the treatment of DFUs is a typical representative of this progress, but the mechanism of action remains unclear. In this review, we integrated bioinformatics and literature and found that ferroptosis is an important signaling pathway through which VSD promotes the healing of DFUs and that System Xc-GSH-GPX4 and NAD(P)H-CoQ10-FSP1 are important axes in this signaling pathway, and we speculate that VSD is most likely to inhibit ferroptosis to promote DFU healing through the above axes. In addition, we found that some classical pathways, such as the TNF, NF-κB, and Wnt/β-catenin pathways, are also involved in the VSD-mediated promotion of DFU healing. We also compiled and reviewed the progress from clinical studies on VSD, and this information provides a reference for the study of VSD in the treatment of DFUs.

## Introduction

Patients with diabetic foot ulcers (DFUs) incur significant hospitalization costs each year, and the ulcers are prone to recurring (1). Vacuum sealing drainage (VSD) was initially used mainly to treat open fractures, but its use has gradually expanded to include the continuous healing of late-stage wounds. This treatment is now widely used in many clinical disciplines, such as general surgery, peripheral vascular surgery, burn plastic surgery, and orthopedic surgery, among others, and shows satisfactory clinical results (2). Although VSD is widely used in surgery, the mechanism of action of VSD in the healing of DFUs remains not fully understood; thus, it is important to study the molecular mechanism of action of VSD in DFU healing (3).

Because diabetic patients suffer from long-term metabolic disorders, especially elevated blood glucose levels and significantly reduced autoimmunity, these patients are highly susceptible to vasculopathy, which leads to ischemia and hypoxia in distal tissues (4). After ischemia or hypoxia, the antimicrobial capacity and bactericidal function of local tissues are significantly reduced (5). In addition, the ability of local leukocytes to phagocytose bacteria is also significantly reduced, and due to the accumulation of pathogenic microorganisms after local ulceration, the tissue becomes highly susceptible to infection (6). An individual with diabetes is also prone to neuropathy (7). After neuropathy occurs, the protective response produced by the body is weakened, and the patient becomes vulnerable to injury; thus injury decreases the degree of self-awareness, which may lead to delays in wound treatment and increase the likelihood of infection (8). These three factors are the main reasons explaining why patients with diabetes are prone to infection. The accumulation of advanced glycosylation end products (AGEs) at the latter stages of diabetes accelerates the onset of diabetic complications, particularly DFUs. All DFUs are contaminated wounds, and control of this infection thus requires long-term application of antibiotics, which causes the body to produce a variety of drug-resistant bacteria (9). In general, antibiotic resistance is increasing, and the available antibiotics need to be improved. Severe sepsis can occur, infections are difficult to control and life-threatening, and the discovery of a drug or technology that can control infection and promote healing is urgently needed (10).

Due to increased interdisciplinary research, VSD has been used in clinical medicine; from a material science perspective, VSD is performed with polyethylene drainage tubes using hydrated alcohol alginate foam dressings to cover or fill skin tissue defects (11). The biological semipermeable membrane is used to seal the wound, and the drainage tube is then connected to a negative-pressure pump to control external bacterial invasion by controlling the negative pressure (12). The polyvinyl alcohol (PVA) hydrated alginate foam dressing used in VSD is white, nontoxic, and nonimmunoreactive; shows high adsorption and permeability; is resistant to corrosion; and consists of a soft texture with high tensile strength (13, 14). Table 1 provides the full list of abbreviations in English.

**Table 1:** Full definitions of the abbreviations in the article (according to the alphabetical order).

Abbreviated name	Full name
AGEs	Advanced glycosylation end products
ACSL4	A synthase long chain family member 4
ALOX15	Arachidonate 15-lipoxygenase
BCL-2	B-cell lymphoma-2
CXCL3	Recombinant chemokine (C-X-C motif) ligand 3
CXCL8	Recombinant chemokine (C-X-C motif) ligand 8
CoQ10	Coenzyme Q 10
CoQ	Coenzyme Q
DFUs	Diabetic foot ulcers
DEGs	Differentially expressed genes
EMT	Epithelial-to-mesenchymal transition
ECM	Extracellular matrix
MEK/ERK	Extracellular regulatory protein kinase
Fpn-1	Ferroportin 1
Fpn	Ferroportin
FSP1	Ferroptosis suppressor protein 1
Fer-1	Ferrostatin 1
Frz	Cell surface receptor frizzled
Fzd	G protein-coupled receptor
GSH	Glutathione
GPX4	Glutathione peroxidase 4
HO-1	Heme oxygenase-1
HMOX1	Recombinant human heme oxygenase 1
IL-1β	Interleukin 1β
IL-1	Interleukin 1
IL-2	Interleukin 2
IL-6	Interleukin 6
IL-10	Interleukin 10
IGF-1	Insulin-like growth factor-1
IκB	Inhibitor of NF-*κ*B
IKK	IκB kinase
IKKα	Inhibitor kappa B kinase *α*
IKKβ	Inhibitor of nuclear factor kappa B kinase subunit beta
KEGG	Kyoto Encyclopedia of Genes and Genomes
LRP5/6	Low-density lipoprotein receptor-related protein 5/6
LPCAT3	Lysophosphatidylcholine acyltransferase 3
MDA	Malondialdehyde
MMPs	Matrix metalloproteinases
MMP1	Matrix metalloproteinase 1
MMP2	Matrix metalloproteinase 2
MMP3	Matrix metalloproteinase 3
MMP9	Matrix metalloproteinase 9
NF-κB	Nuclear factor kappa B
NAD(P)H	Nicotinamide adenine dinucleotide phosphate
NCOA4	Nuclear receptor coactivator 4
PRNP	Prion protein
PCBP1	Poly(rC)-binding protein 1
PCBP2	Poly(rC)-binding protein 2
PLOOH	Phospholipid hydroperoxide
PVA	Polyvinyl alcohol
ROS	Reactive oxygen species
RTAs	Radical-trapping antioxidants
SLC7A11	Solute carrier family 7 member 11
Steap3	Six-transmembrane epithelial antigen of prostate 3
TGF-β	Transforming growth factor β
TGF	Transforming growth factor
TI	Transferrin
Tf-Fe	Transferrin-bound iron
TfR	Transferrin receptor
NF-κB	Nuclear factor kappa B
TNF	Tumor necrosis factor
TNF-α	Tumor necrosis factor α
TIMPs	Tissue inhibitor of metalloproteinases
TIMP1	Tissue inhibitor of metalloproteinase 1
VEGFs	Vascular endothelial growth factors
VEGF	Vascular endothelial growth factor
VSD	Vacuum sealing drainage
VEGFRs	Vascular endothelial growth factor receptors
Wnt	Wingless type
Wnt/β- catenin	Wingless type/β-catenin
WNT 5a	Wingless type 5a
WNT16	Wingless type 16
Xc	System Xc

## Molecular mechanism

**Figure 1:**
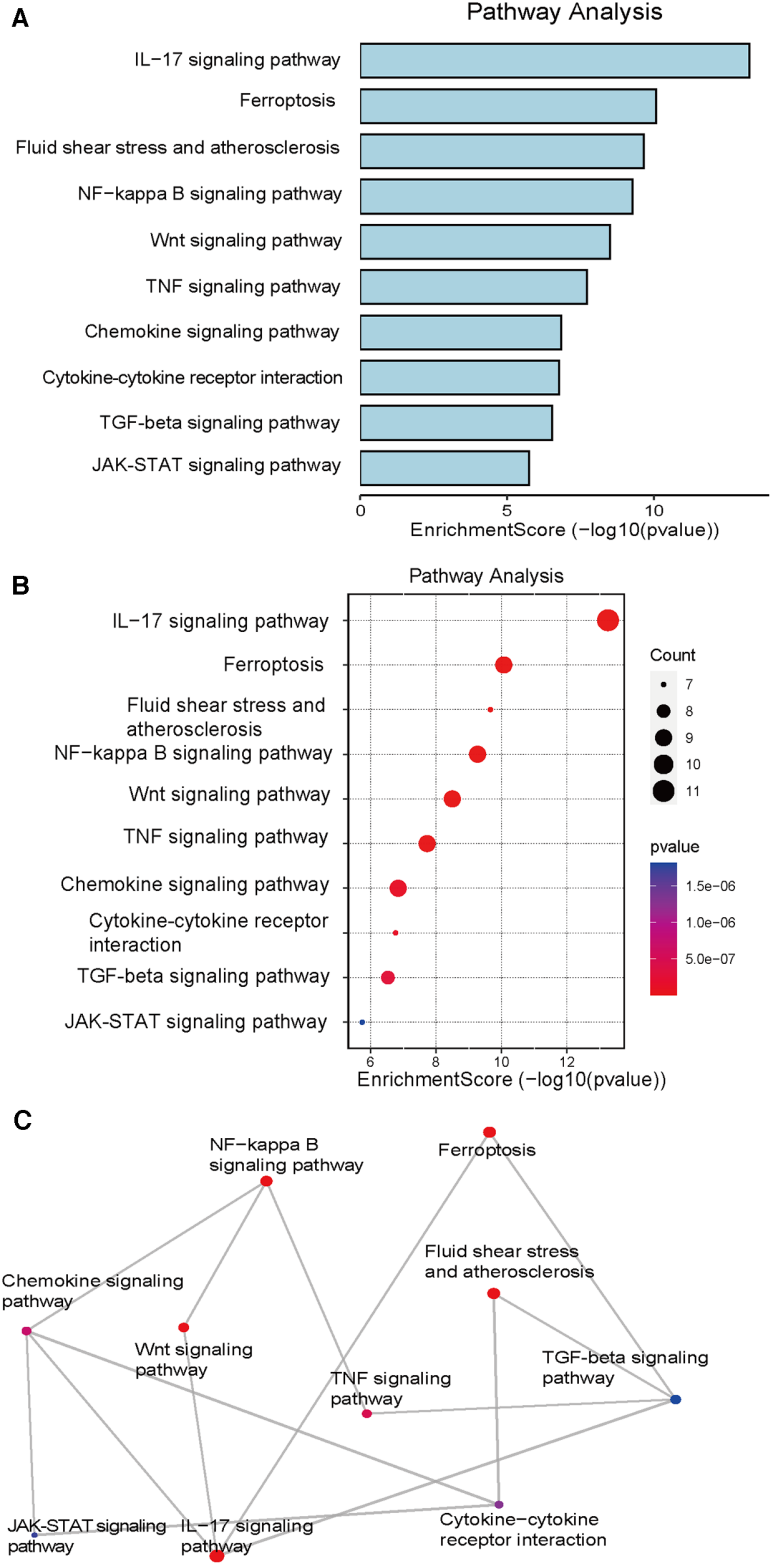
KEGG pathway analysis. (**A**) The bar graph shows the summarized significant signaling pathways ranked by the number of genes present in each signaling pathway. (**B**) Significant KEGG pathway enrichment of DEGs. The pathways are ranked by the number of enriched genes. (**C**) Mesh diagram showing common gene intersections occurring in meaningful signaling pathways. DEGs, differentially expressed genes; KEGG, Kyoto Encyclopedia of Genes and Genomes.

We performed a bioinformatics analysis of the gene expression profiling of tissues before and after VSD treatment of DFUs and combined the results with those detailed in literature reports of the application of VSD in the treatment of DFUs. These data were integrated to identify the following four important molecular mechanisms that are representative of the mechanism of action of VSD in DFUs: ferroptosis, TNF, NF-κB, and Wnt/β-catenin. Interestingly, we found that the involvement of the ferroptosis mechanism in the application of VSD in the treatment of DFUs has been less studied, and we thus focused on the ferroptosis mechanism. A bar graph (Figure 1A) of the KEGG signaling pathways involved in the activity of VSD in the healing of DFUs was analyzed using bioinformatics techniques, and ferroptosis, TNF, Wnt/β-catenin and NF-κB were the top-ranked pathways. Figure 1B shows a scatterplot of the KEGG signaling pathways. We filtered the top-ranked signaling pathways and plotted the number of overlapping genes between different signaling pathways in Figure 1C to visualize the network diagram, and a higher number of crossing lines indicates a greater number of overlapping genes.

## Ferroptosis

Iron is the most abundant and indispensable trace element in the body and is involved in a variety of important physiological and biochemical bodily functions. For example, iron is the main raw material in hemoglobin and myoglobin synthesis. This element is not only involved in the biosynthesis of DNA and ATP but also an important cofactor in the electron transport chain in mitochondria and in many redox reactions of important enzymes (15). Iron is also a cofactor of many enzymes in mitochondria. The body maintains a dynamic balance of iron through dietary iron intake and the “iron cycle”. Hepcidin, which is synthesized and secreted by the liver, directly regulates the serum iron levels, whereas iron homeostasis in the body's cells is mainly regulated via the iron-regulating protein system, which includes the membrane iron transporter ferroportin-1 (Fpn-1) (16). Abnormalities in the distribution and content of iron in the body can lead to the occurrence of various injuries and diseases.Our bioinformatics analysis of the sequencing results revealed that DFUs treated with VSD show an increase in PCBP2 and a decrease in NCOA4 (17, 18), which is a related protein that mediates ferritin-associated autophagy and plays an important role in ferroptosis (19–21).

PCBP1 and PCBP2 are cytoplasmic iron chaperones. Ferroptosis is closely related to iron ions, and these factors are responsible for the transport of iron to ferritin for iron metabolism, storage, and transit (22). In vivo, trivalent iron ions enter the bloodstream and bind to transferrin (TI) to form transferrin-bound iron (Tf-Fe), and most Tf-Fe is used in hematopoiesis of erythrocyte precursor cells. Tf-Fe binds to transferrin receptor (TfR) on the surface of the cell, and the membrane iron transport protein ferroportin (Fpn) is the only iron-transferring protein that attenuates iron death by reducing the intracellular iron levels (23). Iron channels can only transport divalent iron ions into the cytoplasm, and iron ions are reduced from trivalent to divalent; Steap3 acts as a metal reductase to regulate ferroptosis during this process. Our bioinformatics analysis showed that Steap3 expression in DFU tissues is high and decreases after VSD treatment (24, 25).

**Figure 2:**
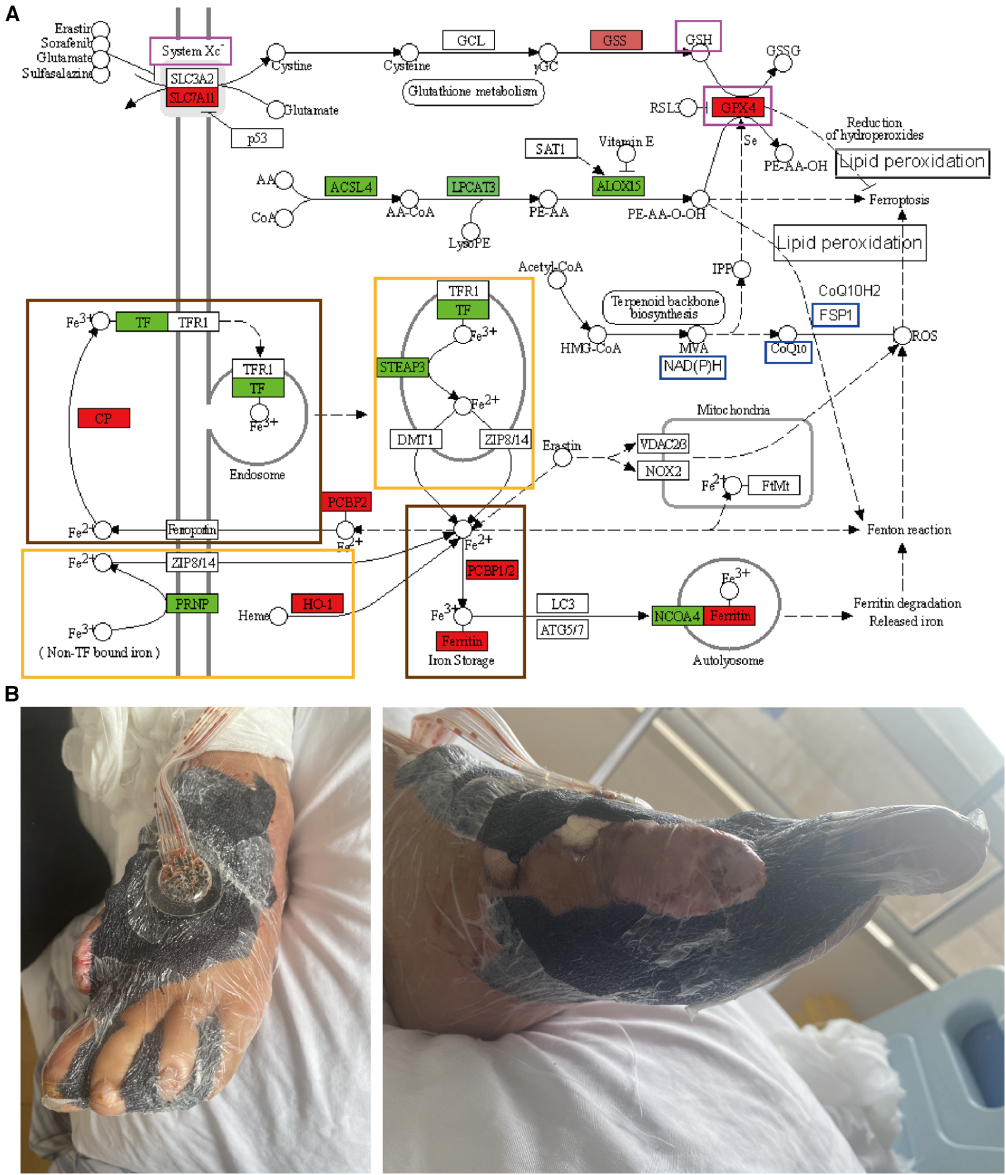
Ferroptosis signaling pathway and photographs of the clinical treatments. (**A**) Ferroptosis is an effective signaling pathway by which VSD can treat DFUs. Genes labeled in green show decreased expression after VSD treatment of DFUs and generally promote iron death; genes labeled in red exhibit increased expression after VSD treatment of DFUs and generally inhibit iron death. The three genes in the purple frame comprise the first iron-death pathway (System Xc-GSH-GPX4), and the three genes in the blue frame form the second iron-death pathway (NAD(P)H-CoQ10-FSP1). The two large frames in brown indicate the conversion of Fe^2+^ to Fe^3+^, and the two large frames in yellow indicate the conversion of Fe^3+^ to Fe^2+^. (**B)** Photographs of DFUs treated with VSD.

Studies have shown that intracellular membranes and the plasma membrane, which contain polyunsaturated fatty acids, are particularly susceptible to peroxidation by lipid radicals, and Fe^2+^ can markedly increase this reaction rate (26). Higher levels of iron ions are associated with a more intense lipid peroxidation reaction and more severe cell damage. Studies of neurons found that the levels of iron ions are positively correlated with the levels of lipid radicals, malondialdehyde (MDA) and other peroxidation products (27). These findings suggest that iron and reactive oxygen species (ROS) initiate and mediate ferroptosis and that the activity of neurons is negatively correlated with the production of lipid peroxidation products (28). When tissue is damaged or exposed to ischemic or hypoxic conditions, cellular metabolism is impaired, and the pH value decreases; these changes result in the reduction of intracellular Fe^3+^ to Fe^2+^, which promotes the production of oxygen radicals with hydrogen peroxide through the Fenton reaction and a rapid reaction with neighboring molecules *in vivo*, and during this process, the lipid components of the cell are peroxidized to generate many lipid radicals (29). The conversion between Fe^3+^ and Fe^2+^ is shown in Figure 2A. Because the cell and plasma membranes are enriched with polyunsaturated fatty acids, lipid radicals induce a cascade reaction, which leads to further thinning of the cell and plasma membranes and causes a barrier loss effect (30). This change explains the thinning of the superficial fascial layer observed in a previous study. Specifically, due to the barrier loss effect, the intracellular ROS levels are increased, damaging the cytosol, inducing protein pore formation in cell membranes, including the plasma membrane, disrupting intracellular homeostasis (31) and inducing more consequential biochemical reactions. In contrast, lipid free radicals damage the lipid structure of the cell and produce lipid peroxidation products, which continuously destroy the cell and eventually lead to irreversible destruction of the structure and function of the cell and plasma membranes via iron-dependent lipid peroxidation-based cell death, i.e., ferroptosis (32). A review of the literature revealed that ferroregulin is a key negative regulator of iron metabolism in the body and binds to the target protein membrane transferrin for its degradation and that FPN is the only known protein with an iron efflux function (33, 34).

Pathways such as the glutathione (GSH) pathway have been proposed to promote cellular keratinization and ulcer healing, and the presence of ferroptosis in DFUs has been demonstrated (35). The GSH pathway is a key pathway regulating oxidative stress damage, and cell death is thought to be associated with DNA and mitochondrial damage due to peroxidation. Dixon (36) introduced the concept of ferroptosis, a novel form of programmed cell death that depends on lipid peroxidation driven by a process that requires intracellular enrichment with available iron (36, 37). The morphology of ferroptotic cells is characterized mainly by changes in the mitochondrial morphology and cristae but no changes in the nucleus (38). Biochemically, ferroptosis is characterized by elevated iron ion levels, high levels of ROS production, decreased glutathione peroxidase 4 (GPX4) activity, and the accumulation of lipid metabolites. Ferroptosis differs from apoptosis, cell necrosis, and autophagic cell death (39). Bioinformatics studies conducted by our research group revealed elevated expression of GSH and GPX4 in tissues after the treatment of DFUs with VSD. The main mechanism of ferroptosis is the induction of cell death by the catalyzed lipid peroxidation of highly expressed unsaturated fatty acids on cell membranes in the presence of divalent iron or ester oxygenase; in addition, ferroptosis is manifested by a decreased level of the regulatory core enzyme GPX4 in the antioxidant system (glutathione system) (40). Numerous studies have shown that ferroptosis is closely associated with neoplastic diseases, neurodegenerative diseases and organ damage (41). Stress therapy can promote GSH synthesis by stimulating SLC7A11 (system Xc) expression in mice with DFUs (42), improve GPX4 activity, protect cells from oxidative stress, maintain the cellular redox balance, and attenuate ferroptosis by reducing lipid peroxidation (17, 43). Our bioinformatics study revealed elevated expression of System Xc in DFU tissues after VSD treatment.

Ferroptosis is thought to be inhibited only by the phospholipid hydroperoxide (PLOOH) reductase GPX4 and free radical-trapping antioxidants (RTAs) (44). However, identifying factors that sensitize specific cell types to ferroptosis is important to gain a greater understanding of the pathophysiological role played by ferroptosis and how it can be leveraged in the treatment of trauma-induced lesions. Although metabolism-limiting factors and phospholipid components promote cell susceptibility to ferroptosis, no cellular mechanisms that cause ferroptosis resistance *per se* have been identified (45). The unique triggering mechanism of ferroptosis implies its potential application in the treatment of tumors (46). Due to continuous research into the mechanism of ferroptosis, acetyl coenzyme A synthase long chain family member 4 (ACSL4) has been identified as a ferroptosis-promoting gene, and its expression level determines the sensitivity of cells to ferroptosis to some extent. However, certain cancer cells are insensitive to GPX4 inhibitors regardless of whether ACSL4 is expressed, which suggests that other ferroptotic regulatory mechanisms are not dependent on GPX4 (40). A bioinformatics screening of genes revealed that stress therapy decreases the levels of ALOX15, ACSL4, and LPCAT3 in diabetes mouse wound models (47, 48). The literature suggests that the accumulation of ALOX15, ACSL4 and LPCAT3 promotes ferroptosis (17, 49).

Iron chelators (desferrioxamine) and antioxidants (*α*-vitamin E, β-carotene, etc.) have been shown to significantly inhibit erastin-induced ferroptosis, which suggests that iron homeostasis and lipid peroxidation are key links in ferroptosis (28). Dixon (50) and Stockwell (51) suggested that the iron in iron chelators is a coenzyme for many important metalloenzymes and prevents the transfer of electrons from iron to oxide molecules, which inhibits the production of oxygen radicals, i.e., inhibits cellular lipid peroxidation and thus ferroptosis. One ferroptosis inhibitor, ferrostatin-1, is a lipid ROS scavenger with an N-loop that acts as a lipophilic anchor to biological membranes but does not inhibit the MEK/ERK pathway or chelate iron (28, 52). Scholars have performed protein sequencing analysis of DFU tissues collected before and after VSD treatment and found that the treatment decreased the expression of HO-1 and PRNP and increased that of GPX4 (17, 53, 54). A KEGG pathway analysis revealed that these factors are closely related to ROS and ferroptosis (55, 56).

In addition, Doll S et al. (57) and Bersuker K et al. (58) discovered a ferroptosis regulator, ferroptosis suppressor protein 1 (FSP1), independent of the GPX4 system. FSP1 inhibits ferroptosis by reducing the CoQ10 level and thus blocks lipid peroxidation. The NAD(P)H-CoQ10-FSP1 pathway is another key mechanism of lipid peroxidation and ferroptosis inhibition. Thus, lipid redox homeostasis can be separated from the classical GSH-GPX4 mechanism and forms a relatively independent mechanistic branch. Moreover, compared with the lethal consequences of complete GPX4 knockout, mice with complete FSP1 knockout do not show obvious visible morphological or developmental abnormalities, which indicates that FSP1 is merely a powerful synergistic partner of GPX4 and cannot perform the key regulatory function of GPX4 in ferroptosis. Our bioinformatics analysis found that the expression of FSP1 and CoQ10 is elevated in tissues after the treatment of DFUs with VSD. Therefore, the application of GPX4 inhibitors is necessary in future ferroptosis-based DFU treatment strategies, and the use of appropriate means to inhibit synergistic proteins such as FSP1 is also important (58).

Ferroptosis is characterized by lipid peroxide-dependent accumulation in the pathogenesis of diabetic wounds, and fibroblasts and vascular endothelial cells are thus exposed to high concentrations of glucose *in vitro*, harbor high levels of ROS, lipid peroxidation products, and ferritin, and thus exhibit reduced proliferation and migration rates (59). These effects of a high glucose concentration are significantly reduced after treatment with ferrostatin-1 (Fer-1), a ferroptosis inhibitor; in a diabetic rat model, the direct application of Fer-1 to wound sites reduces oxidative stress and inflammatory marker expression, which accelerates wound healing. Ferritin formation has been associated with wound healing and is a potential new therapeutic target in difficult-to-treat diabetic wounds; that is, the attenuation of ferroptosis in diabetic wounds may promote the healing of DFUs (60). Persistent inflammatory and oxidative stress at a wound site is a clear manifestation of delayed wound healing in DFUs, and chronic hyperglycemia in patients can lead to the accumulation of lipid peroxidation products in the circulatory system and impaired iron metabolic pathways (61), resulting in multiple free iron species in plasma. Oxidative stress and lipid peroxidation are typical manifestations of ferroptosis in DFUs, and ferroptosis is one of the potential mechanisms involved in delayed wound healing in DFUs; thus, ferroptosis may be a new therapeutic target of delayed wound healing in DFU patients (62). Through *in vivo* experiments, a high glucose concentration-induced cell injury model has been established, and the expression levels of ferroptosis-related proteins and the related inflammatory cytokines IL-1β and IL-10 have been measured, confirming the presence of cellular ferroptosis in DFUs and its effect on DFU healing (63). In addition to *in vivo* experiments, *in vitro* cellular experiments have been established with vascular endothelial cells and fibroblasts (64). The results showed that ferroptosis is evident in DFUs and that inhibition of ferroptosis improves the activity of fibroblasts and vascular endothelial cells exposed to high glucose concentrations and thus improves cell migration and proliferation (62, 65). At the cellular level, Fer-1 ameliorates high glucose- and high fat-induced lipid peroxidation and affects ROS production. More importantly, HMOX1 knockdown attenuates Fe^2+^ overload, reduces the iron and ROS levels, and alleviates lipid peroxidation, which results in reduced iron ion accumulation in diabetic endothelial cells (66). The upregulation of HMOX1 is critical for increased ferroptosis during the development of diabetic atherosclerosis, suggesting that HMOX1 may serve as a potential therapeutic or drug development target in diabetic atherosclerosis (67). DFUs generally develop over years of poor glycemic control in diabetes, which causes peripheral neurovascular lesions in the lower extremities (68). DFUs generally develop as follows: years of poor glycemic control cause peripheral neurovascular disease in the lower extremities, and this disease is further aggravated by the development of atherosclerotic occlusions (69), which lead to localized ischemia and hypoxia in the foot and ultimately cause ulceration. Ferroptosis is caused by unrestricted lipid peroxidation, diabetes mellitus is closely related to atherosclerosis, and DFUs are closely related to ferroptotic, lipid peroxidation, and atherosclerotic pathways (67, 70). The molecular mechanisms are shown in Figure 2A, which reveals that ferroptosis is the most highly enriched molecular mechanism identified by bioinformatics, and the genes that usually promote ferroptosis are labeled green. The analysis of the tissue sequencing results showed that the expression of these genes decreased after the treatment of DFUs with VSD and that VSD inhibited ferroptosis to promote the healing of DFUs by reducing the expression of the green-labeled genes. The red-labeled genes, which are genes that usually inhibit ferroptosis, showed an increase in expression after the VSD treatment of DFUs, and VSD inhibits ferroptosis by promoting high expression of the red-labeled genes to promote DFU healing. Fe^2+^ and Fe^3+^ can be converted to each other. System Xc-GSH-GPX4 and NAD(P)H-CoQ10-FSP1 are the two axes through which VSD promotes DFU healing. Ferroptosis may be a new signaling pathway for the future study of the application of VSD for DFUs, and system Xc-GSH-GPX4 and NAD(P)H-CoQ10-FSP1 may be a new axis for the future study of the application of VSD for DFUs.

## TNF

The TNF signaling pathway is closely related to matrix metalloproteinases (MMPs) and plays a central role in orchestrating the inflammatory immune response, is involved in the intervention of chronic inflammation and autoimmune diseases, and plays an important role in the repair of DFUs (71). Collagen is essential for wound healing, and MMPs are the main enzymes that degrade collagen. In contrast, metalloproteinase inhibitors inhibit MMP activity and block extracellular matrix degradation (72). The gene expression and activity of MPP-3 in DFU injury exudates and tissues are reduced after VSD, suggesting that VSD could accelerate DFU healing by inhibiting the gene expression and activity of MMP-3 and increasing the gene expression of metalloproteinase inhibitors (73). VSD also increases the expression of VEGF, IGF-1, and BCL-2 in DFUs, inhibits the expression of C-FOS, stimulates vascular endothelial cell proliferation and inhibits apoptosis (74).

VSD can reduce the levels of MMP-2, increase the levels of VEGF and TIMP-1, and promote the healing of ulcer wounds in patients with DFUs (75). Recent studies have shown that MMPs constitute the main family of enzymes involved in the degradation of the extracellular matrix (ECM) and are involved in the extracellular degradation and breakdown of matrix proteins during normal physiological processes such as wound healing and vascular proliferation, which prompts cells to cross the basement membrane (76). The ECM is closely related to fibroblasts, keratin-forming cells, cell proliferation and migration, and hair regeneration, among others. Changes in and remodeling of the ECM and the combined actions of multiple matrix-degrading enzymes and their inhibitory enzymes make ECM proteolysis the key process in wound repair (77). Scholars have shown that 2 weeks of VSD treatment for DFUs significantly decreases the levels of MMP-1, MMP-3 and MMP-9 and significantly increases the levels of TIMP-1 (55). Recent studies have found that MMP-3, which is also known as stromelysin-1 or progelatinase, belongs to the MMP family of stroma-degrading enzymes and is a Zn^2+^-dependent protease (78). MMP-3 degrades a variety of ECM components and is involved in physiological and pathological processes such as tissue morphogenesis, wound repair, and inflammatory responses, and its hydrolytic substrates include type III, IV, V, and IX collagen, proteoglycans, fibronectin, laminin, and elastin. The expression of MMP-3 can be observed at different periods after tissue trauma, and some studies have shown that MMP-3 expression can be detected even 28 days after trauma, which indicates that MMP-3 is involved not only in extracellular matrix degradation but also in wound repair, extracellular matrix remodeling and tissue reconstruction. Tissue inhibitors of metalloproteinases (TIMPs) constitute a group of low-molecular-mass proteins that are found extensively in tissues and bodily fluids and form on endothelial, fibroblast, and epithelial cell basolateral membranes (79). Endogenous inhibitors of MMP-1, such as TIMP-1, specifically inhibit matrix-degrading enzymes in MMP-1. In addition, TIMP-1 is a multifunctional molecule that not only exerts cell growth factor-like effects but also promotes fibroblast proliferation and collagen synthesis, which results in deposition of the ECM, inhibition of its degradation, regulation of ECM metabolism and inhibition of vascular degradation. TIMP-1 regulates ECM metabolism and inhibits angiogenesis. In contrast, cytokines, growth factors and hormones regulate the expression of MMP-1 and TIMP-1 through complex pathways (80). For example, MMP-3 regulates interleukin-1 activity, and in contrast, the inhibitory enzymes activated by MMP-3 can inactivate cytokines; thus, MMP-3 may be involved in the regulation of interleukin-1 activity in chronic inflammation and thus affects delayed DFU healing or increases fibrosis. Based on a combination of bioinformatics results with the literature, we found that 2 weeks of the treatment of DFUs with VSD significantly decreased the levels of IL-1β, TNF-α, MMP-1, and MMP-9 and significantly increased the levels of VEGF, TGF-β1, and TIMP-1 (55).

## NF-κB

The NF-κB family comprises transcription factor proteins that bind to inhibitor of NF-κB (IκB) in the cytoplasm in a resting cellular state (81). IκB is not transcriptionally active. Upon external stimulation, IκB kinase (IKK) is activated to cleave the IκB protein from NF-κB and degrade it (82). NF-κB then enters the nucleus and binds to specific DNA targets in the nucleus, initiating the transcription and expression of target genes, and inflammatory factors such as interleukin-2 (IL-2) and IL-6 are subsequently released from the cell (83). The NF-κB pathway is central to the regulation of the immune response, stress response, apoptosis and inflammation, is a common pathway in many inflammatory processes and plays an important role in tissue repair (84). Bioinformatics results combined with the literature shows that VSD could improve the expression of CXCL8 and CXCL3 in patients with DFUs and promote vascular regeneration and recovery in patients with DFUs by increasing gene expression (85, 86). CXCL3 and CXCL8 act through the NF-κB signaling pathway (87, 88).

The NF-κB pathway is a classical pathway in the inflammatory response, and during wound healing, the NF-κB pathway is immediately activated as an initial immune response and mediates the release of inflammatory factors necessary for wound healing (89). In DFUs, sustained activation of the NF-κB pathway leads to increased secretion of inflammatory factors and contributes to the chronic refractory nature of DFUs, whereas inhibition of the NF-κB pathway accelerates healing (90). Shibata et al. (91) showed that VEGFs/VEGFRs may be downstream target genes in the NF-κB signaling pathway and that NF-κB mediates angiogenic capacity. A study on the promotion of DFU healing revealed that VSD accelerates diabetic wound healing by activating the NF-κB pathway to stimulate macrophage VEGF secretion, which in turn acts on vascular endothelial cells to promote neovascularization (92, 93).

## Wnt/β-catenin

The Wnt signaling pathway is a highly conserved signaling pathway, and the Wnt protein family is involved in many biological processes, including cell proliferation, apoptosis and differentiation, and in the maintenance of stem cell multipotency (94). Three major pathways are involved in Wnt signaling, and the most studied of these pathways is the Wnt/β-catenin pathway, which is also known as the classical Wnt pathway. The Wnt/β-catenin pathway can regulate inflammatory and immune responses by interacting with the NF-κB pathway; similarly, the NF-κB pathway can interfere with the activation of the Wnt/β-catenin signaling pathway (95, 96). The Wnt/β-catenin pathway is involved in DFU healing, which may involve the regulation of inflammation, impacts fibroblasts and plays a role in angiogenesis (97). Using bioinformatics techniques, we found that VSD improves DFU healing by downregulating Wnt4 through activation of the Wnt signaling pathway (98, 99). When the Wnt signaling pathway is activated by VSD, the Wnt protein binds to Frz and LRP5/6 (93).

Bioinformatics results combined with the literature revealed that the treatment of DFUs with VSD induced changes in the Wnt pathway and decreased the expression of Wnt16 and Wnt5a (99, 100). Studies have identified many important functions of Wnt5a, which, upon binding to receptors, activates the Wnt/β-catenin pathway and plays a key role in DFUs. For example, the expression of Wnt5a in DFUs is high and decreases after VSD treatment. During the early stages of wound repair, Wnt5a expression is enhanced in macrophages, and Wnt5a regulates inflammatory factors through its ligand Fzd (101). Wnt5a activates both classical and nonclassical Wnt pathways (102). Fibroblasts produce ECM, which provides structural support for cell growth and nutrient transport (103). Studies have confirmed that β-catenin is specifically involved in the proliferative phase of DFU repair (104). Additionally, it is believed that β-catenin regulates fibroblast motility and is an important regulator of fibroblasts during the proliferation phase of DFU repair (105).

## Scope of the application of VSD in clinical research and precautions that should be considered

When applied to wounds as components of VSD, polyethylene foam and semipermeable polyurethane film create a moisturizing dressing that serves to provide favorable environmental conditions (Figure 2B). We summarized the indications, contraindications, optimal time points, and wound staging of VSD for DFUs. VSD can be applied in the following conditions: after the DFU wound infection is controlled; when DFUs are combined with bone and tendon exposed wounds (106, 107); when DFUs exhibit poor blood flow or a greater risk of flap grafting, when DFUs have a small area of exposed bone and/or tendon; when DFUs exhibit skin line grafting (108, 109); for delayed flaps such as after flap transfer surgery when a longer period is needed to break off the tip of the flap; for dermal substitute grafting (110); during postamputation or toe surgery for first-phase suture wounds; for stump wounds after amputation, after risk and infection control, and after necrotic tissue is largely removed (111–113).

Contraindications to the use of VSD for the treatment of DFUs include the following: DFU wounds with large amounts of necrotic tissue; DFU wounds with active hemorrhage (114); uncovered blood vessels and nerves; DFUs with anaerobic or fungal infections (115); DFUs with periwound skin eczema; and DFUs with osteomyelitis (116, 117). VSD is not suitable for some patients on long-term anticoagulant medications because it may exacerbate the risk of bleeding and should thus be considered with caution (118–120).

The time point and staging of the application of VSD for the treatment of DFUs are important considerations. In the first phase (from 1 to 3 days), hemostasis and inflammatory responses are induced, and these responses are necessary for the removal of microorganisms and tissue debris and are characterized by the infiltration of neutrophils early during this phase and the differentiation of monocytes later during this phase. The second phase (2–10 days) is the proliferative phase, which is characterized by fibroblast proliferation, neovascularization, and granulation tissue formation. The third stage is the repair stage, which can last for 1 year or even longer after trauma and mainly manifests as the contraction of collagen and scarring.

## VSD improves peripheral arterial vascular perfusion and microcirculation in DFUs

Microcirculatory disease in DFUs is not only the pathological basis of secondary neurological and vascular damage in diabetes but also an important factor that affects the treatment and prognosis of patients with DFUs (121). These microcirculatory changes are primarily functional and not structural, and microcirculatory changes manifest as microvascular responses to an impaired vasodilatory capacity (122). An inadequate blood supply to an ulcer site is one of the most important reasons for the persistence of DFUs, and an adequate blood supply is essential for the growth of new granulation tissue at an ulcer site (3). It is also important to note that the diameter of a vessel can be enlarged to reduce the flow rate of red blood cells. Some scholars have approached this topic from a molecular biology perspective and detected increased expression of proangiogenic factors (e.g., VEGF) when the DFU-induced injury surface is under negative pressure (123). Other scholars have used adipose tissue as a filling material for DFUs and have shown that adipose tissue filling in conjunction with negative pressure leads to a synergistic effect with cell regeneration, increasing the expression of TGF and VEGF in local tissue (124). Potter et al. cultured vascular endothelial cells of DFUs *in vitro* and opted to start with intermittent-pressure suction and then switch to continuous pressure suction, and the results showed that intermittent negative pressure was more clinically promising than continuous negative pressure for promoting vascular endothelial cell proliferation and migration (125).

In an animal model of diabetic ulcers treated with VSD, blood flow was measured with an implantable Doppler probe. Compared with the control group, the blood flow velocity was increased in the VSD group, and VSD increased the blood supply to the DFUs and improved perfusion (126–128). In a recent study, Kairinos et al. (129) used a radiotracer technique to measure tissue perfusion in healthy subjects and found a correlation between increased perfusion and decreased negative-pressure values. It is believed that thermal diffusion techniques, corrosion casting, and fluorescent particles will soon be used to gain more insight into the mechanisms of angiogenesis.

## VSD enhances peripheral vascular permeability and reduces tissue edema caused by peripheral arteries

Polykandriotis et al. (130) found that the application of VSD increased the density of lymphatic vessels on the DFU margins and further reduced edema. Consistent with this finding, VSD has been widely reported to reduce DFU-associated edema. VSD can effectively reduce edema in an ulcerated surface and tissues near DFUs, increase vascular permeability, and ultimately accelerate granulation and tissue formation (131). After the application of VSD, lymphocytic component infiltration into the DFU surface has been observed to subside faster, collagen synthesis appears earlier during the proliferative phase, and enhanced contractile fiber synthesis is observed during the repair phase (132). A histological study confirmed that VSD could produce dermis in the margin of DFUs. VSD can induce the transition of superficial vascular endothelial cells and fibroblasts in the dermis at the edge of DFUs into an active proliferative state, increase the density of microvessels and dilate narrow and occluded capillaries (133), and all of these effects are conducive to the repair of DFUs and the formation of granulation tissue.

## VSD maintains wound surface moisture and cleanliness and prevents bacterial growth

Ning et al. (134) found that VSD helps remove necrotic tissue from DFUs and soluble inflammatory mediators that locally inhibit cell proliferation. During the aspiration of ulcer tissue fluid, unfavorable cytokines and enzymes that hinder healing are also removed. VSD promotes proliferation of the lymphatic network in DFU periwound tissue, and the combined effect of these actions can transform DFUs into a positive and conducive environment for wound healing (135). Scholarly research has studied the bacterial concentrations in DFU patients treated with either VSD (treatment group) or saline gauze (control group) and found a difference in these bacterial concentrations between the two groups, with the VSD group showing significantly fewer gram-negative cocci than the saline gauze group (136). These studies show that VSD plays a significant role in controlling DFU infection and reducing the number of bacteria, and research into biofilms and defenses will likely be important research directions in the future (137).

## VSD reduces the inter-tissue pressure and stimulates growth factors

The negative-pressure suction of VSD can reduce the inter-tissue pressure, including the partial pressure of oxygen around a DFU-induced lesion, and stimulate signaling to initiate repair (138). VSD can indirectly affect the levels of repair factors in DFUs via inter-tissue pressure and oxygen partial pressure, which stimulates growth factors and promotes the healing of DFUs. Treatment with VSD has been shown to result in a higher rate of DFU healing, faster healing, and a reduced need for amputation compared with standard therapy (112). VSD therapy is not only safer and more clinically effective than advanced moist-wound therapy in the treatment of DFUs but also provides better control of complications and reduces the likelihood of infection (139). Compared with standard wound dressings, VSD reduces the granulation time of wounds after DFU amputation by 40%, resulting in 90% granulation being achieved within a shorter period and fewer complications (140). The negative pressure of VSD can reduce the pressure between DFU tissues and decrease the partial pressure of oxygen around a wound (141). VSD stimulates signals that initiate repair, accelerates the removal of DFU necrotic tissue, and induces the secretion of fibrin activators (142). Fibrinolysis in DFUs enhances the deposition of collagen and creates an environment that accelerates fibrinolysis, which promotes autolytic debridement (143, 144).

## Revolutionary changes to the VSD system can reduce DFU neuropathy

Dysfunctional vascular endothelial cells and vascular smooth muscle cells cause a reduced vasodilatory capacity in diabetic patients; moreover, microvascular diastolic function associated with neuraxial reflexes is also impaired in diabetic patients, and vasodilation and impaired neuraxial reflexes have been increasingly recognized as important factors that make the healing of secondary ulcers in diabetes difficult (145, 146). VSD expands the outcome of traditional tubular punctiform drainage into a full range of continuous negative-pressure drainage outcomes, which can lead to the timely draining of necrotic tissue debris and inflammatory exudate from DFUs (147), and these effects minimize the accumulation of bacteria and toxic decomposition products produced by necrotic tissue in DFUs and produce a “zero accumulation” state that is more effective for controlling infection (148). Scholars have found that the application of VSD to patients with DFUs improves the nerve conduction velocity and reduces neuropathy in patients and that optimized VSD is more effective in improving neuropathy, which may be related to regulating the level of oxidative stress and promoting vascularization of the DFUs (149). Moreover, closure of the semipermeable adhesive film can isolate the drainage area from the outside environment and thus prevent DFU infection (150). Moist DFUs under an airtight dressing are more easily infiltrated by polymorphonuclear cells than dry DFUs after a traditional dressing is changed and covered by gauze, which provides a good local environment for immune cells to phagocytose and kill bacteria and thus facilitates the prevention of DFU infection and promotes healing (151). Moreover, the mechanical tension produced by continuous high negative-pressure suction can cause the layers of a DFU tissue section, which are always attached to each other, to exert a natural physical pulling force on the edges of the DFUs and promote healing (152), which explains why VSD can transform a chronic DFU into a normally healing wound.

## Conclusion and prospects

VSD is effective in treating DFUs in the clinic, and the literature evaluates the clinical effects of VSD. VSD can improve the peripheral arterial perfusion of DFUs, reduce tissue edema, and increase vascular permeability. The mechanical stress generated by VSD can effectively promote cell proliferation and tissue repair, stimulate the expression of factors for DFU repair, reduce neuropathy, stimulate the growth of granulation tissue, and promote the healing of DFUs.

However, the specific molecular mechanism by which VSD promotes DFU healing is unclear. We performed a bioinformatics analysis of the results from the tissue sequencing of VSD-treated DFUs and integrated the genes and signaling pathways analyzed using bioinformatics techniques with the literature. The resulting review revealed that VSD can promote DFU healing by decreasing the level of ferroptosis and regulating the TNF, Wnt/β-catenin, and NF-κB signaling pathways to promote healing. Ferroptosis is the most highly enriched molecular mechanism identified by bioinformatics. System Xc-GSH-GPX4 and NAD(P)H-CoQ10-FSP1 are two important axes through which VSD promotes healing in DFUs, which may be new signaling axes that should be investigated in the future for the treatment of DFUs with VSD.

The in-depth investigation of the mechanism of action of VSD in promoting DFU healing is very important. However, this work still has many shortcomings; for example, we did not analyze the molecular biology characteristics of DFU wound repair at different stages and did not carry out any further validation by histology. Nevertheless, our work is sufficient to show that VSD is a promising strategy to promote DFU healing and can provide a molecular biology reference.
